# Evolutionary algorithms simulating molecular evolution: a new field proposal

**DOI:** 10.1093/bib/bbae360

**Published:** 2024-08-11

**Authors:** James S L Browning Jr., Daniel R Tauritz, John Beckmann

**Affiliations:** Department of Computer Science and Software Engineering, Samuel Ginn College of Engineering, 3101 Shelby Center, Auburn, AL 36849-5347, United States; Department of Computer Science and Software Engineering, Samuel Ginn College of Engineering, 3101 Shelby Center, Auburn, AL 36849-5347, United States; Department of Entomology and Plant Pathology, Auburn University College of Agriculture, 301 Funchess Hall, Auburn, AL 36845, United States

**Keywords:** artificial intelligence, evolutionary algorithms, computational evolution, computational biology, genetic programming, molecular evolution, proteomics, biotechnology

## Abstract

The genetic blueprint for the essential functions of life is encoded in DNA, which is translated into proteins—the engines driving most of our metabolic processes. Recent advancements in genome sequencing have unveiled a vast diversity of protein families, but compared with the massive search space of all possible amino acid sequences, the set of known functional families is minimal. One could say nature has a limited protein ”vocabulary.” A major question for computational biologists, therefore, is whether this vocabulary can be expanded to include useful proteins that went extinct long ago or have never evolved (yet). By merging evolutionary algorithms, machine learning, and bioinformatics, we can develop highly customized ”designer proteins.” We dub the new subfield of computational evolution, which employs evolutionary algorithms with DNA string representations, biologically accurate molecular evolution, and bioinformatics-informed fitness functions, Evolutionary Algorithms Simulating Molecular Evolution.

## Antecedents of EASME

EAs model evolution, using selection, reproduction, and mutation to find solutions to optimization and design problems. In 2006, Banzhaf *et al.* proposed a bold new idea—that progress in this field had slowed, but that it could continue to improve by incorporating more of the nuances of the natural world [1]. We propose a new approach to solving biological problems, EASME, which will merge computational evolution (CE) with molecular-level bioinformatics. While *in-silico* evolution often purposely abstracts away from nature, EASME encodes the full complexity of molecular evolution. What is novel to the field of CE today is the realized ability to model actual DNA chromosomes, encoding actual genes, and their downstream proteins in the context of realistic fitness evaluations and structure predictions.

## Defining the problem

**Figure 1:**
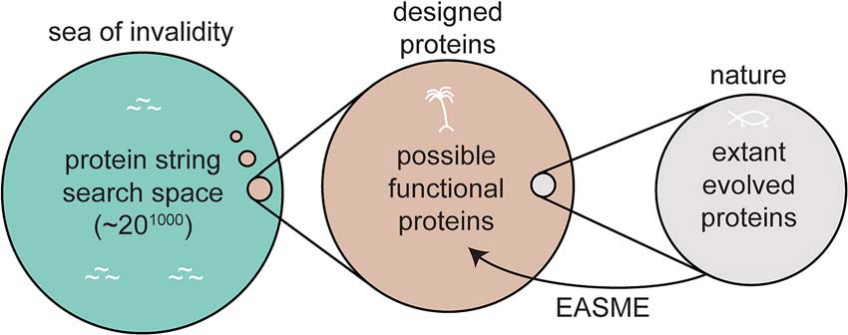
The search space of proteins—a vast sea of invalidity contains a handful of islands containing functional proteins, only a small subset of which have likely been evolved by nature.

Proteomic applications are a primary target for EASME. Proteins are sentences written with an alphabet of 20 amino acids. Many proteins exceed 1000 characters in length, so the search space of possible proteins is vast. Most string permutations would be unstable and non-functional, and thus, the search space of possible proteins exists as a few tiny islands within a vast “sea of invalidity.” Within that sea exists an archipelago of possible functional proteins, and only a small region of those is occupied by the proteins that actually evolved (see Fig. 1). EASME aims to expand the set of extant proteins by colonizing new islands in the sea of invalidity.

The EASME approach employs CE to achieve this end. Recent advancements in machine learning (ML) have led many to claim that complex biological problems, most notably protein folding, are essentially ”solved.” With this being the case, one may wonder why our focus remains on CE. ML does have a place in the pursuit of EASME, but our reasoning for focusing on CE is 2-fold—one, we agree with Banzhaf that models *drawing on* evolution would be the best way to study and fast forward evolution; and two, ML falls short in a key area.

### Where ML falls short

In truth, the advances of ML have not yielded a fundamental understanding of *de novo* protein folding, as was discussed in ”De novo protein folding on computers. Benefits and challenges” by Barry Robson [2]:

...AlphaFold does not solve, or seek to solve, the folding problem. It ”reasons” from what is ultimately biological data, not from fundamental laws of chemical physics.

Importantly, ML models will always be limited by their training sets, which are restricted to the archipelago of extant functional proteins (and usually represent some small subset of those). The current state of many areas of AI could be defined similarly—while astounding advancements have surely been made in the field of deep learning, and this has yielded very impressive results in some cases; these results are ultimately facsimiles of *what* is, not a true understanding of *why* it is.

### Where evolutionary algorithms can help

When it comes to uncovering the *why* of something, evolutionary computation holds a unique advantage. This was succinctly observed in D’Angelo *et al.*’s 2023 paper ”Identifying patterns in multiple biomarkers to diagnose diabetic foot using an explainable genetic programming (GP)-based approach,” in which GP—a type of EA employing complex representations—was used to diagnose diabetic foot [3]. Not only did the team’s GP approach *outperform* ML, but the decisions the program produced were easily comprehensible by human operators.

## Evolutionary algorithms simulating molecular evolution

**Figure 2:**
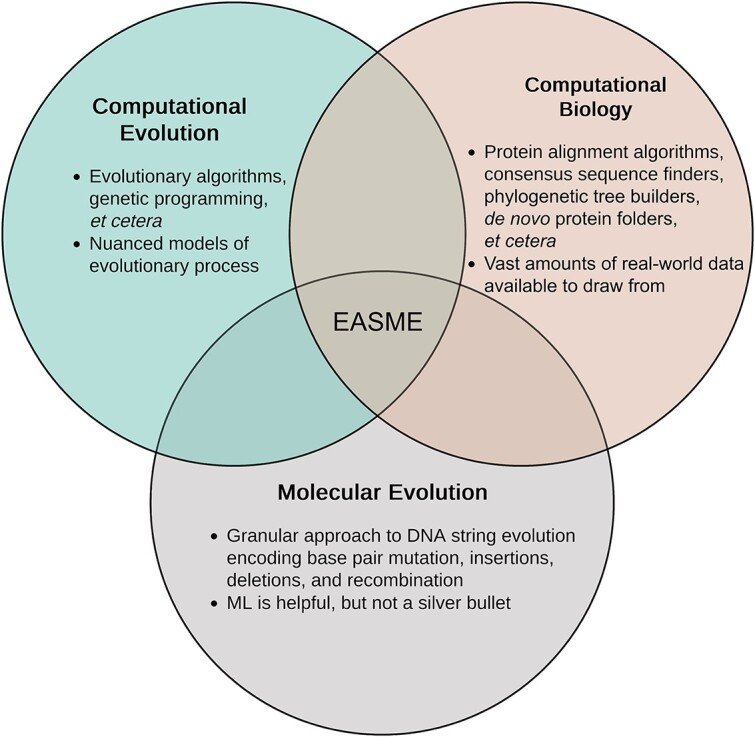
EASME exists at the intersection of CE, computational biology, and molecular evolution, drawing on the strengths of all three to solve problems.

As computing power continues to increase, complex simulations of evolving biochemical systems are approaching feasibility, even on desktop computers. This has been demonstrated most recently in “Modeling the emergence of *Wolbachia* toxin antidote protein functions with an evolutionary algorithm” [4], a project in which researchers drove forward evolution of two specific, interacting proteins involved in cytoplasmic incompatibility. This project specifically modeled a simple two-protein network, rewarding tight binding interactions between a toxin and antidote. That paper provides proof of concept that more complicated protein networks might be modeled and cascading co-evolutionary effects and evolution of novel domains can be tracked within those systems. In another article, ”Molecular dynamics simulation of an entire cell” by Stevens *et al.*, an entire minimal cell was simulated *in silico* [5]. With the computing power to simulate ever more complex biochemical systems and protein–protein interactions now at our disposal, we see a clear path toward modeling protein–protein co-evolution on the level of 2–100 discrete protein interactions, provided that each individual interaction can be expressed in code. Furthermore, through the continued development of this burgeoning field—leveraging the biomimicry of EAs, ML, and state-of-the-art hardware (see Fig. 2)—we discern that uncovering fundamental grammar structures and syntax of our DNA and proteins is now possible and that generative AI in the form of EASME can rewrite novel useful proteins.

## The path forward

**Figure 3:**
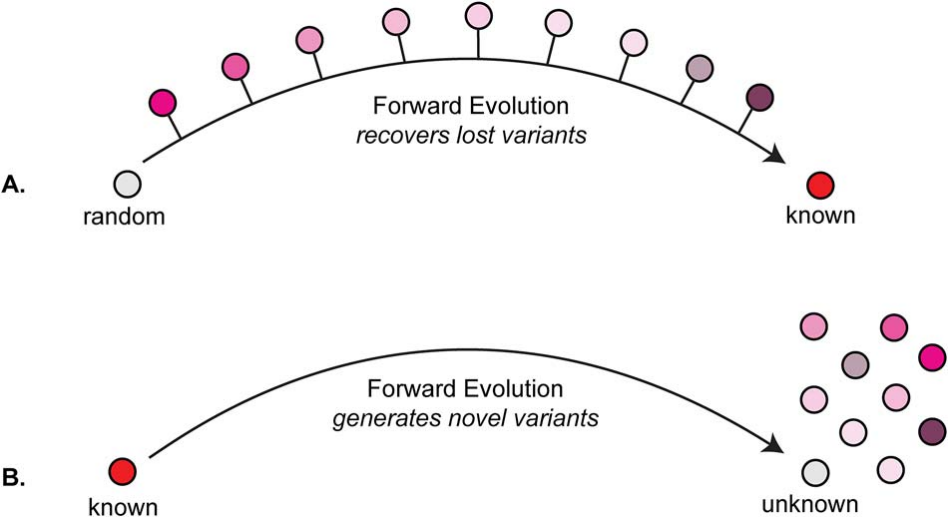
Ways to employ EASME.

EASME represents a specific effort to focus on expanding the set of extant proteins. The core EASME algorithm, once developed, can run in a few distinct ways (see Fig. 3). First, EASME can evolve a random sequence toward a known consensus sequence (“*unknown to known*”). In this context, the desired outcome is to reconstruct sequence clusters that went extinct during the process of evolution. Selective fitness is implemented by pushing the evolution toward a known protein sequence family. EASME outputs samples of Pareto optimal sequences from theoretical evolutionary intermediates, effectively recovering extinct sequence variants. How much the EASME generated sequences would differ from real historical intermediates is unknowable without ancient genomes. However, the utility of generated sequences can be tested, measured, and linked to a corresponding successful discovery rate. The second way to run EASME is *known to unknown*, where a known entity is forward evolved into the future by implementing a selection regimen that drives toward a desired characteristic phenotype. This methodology outputs Pareto optimal sequences that may have never evolved yet and is effectively a fast forward button on evolution into the future. While this approach would undoubtedly produce many false positives, wet lab work will allow us to test and validate designed proteins while simultaneously honing a given enzyme’s fitness function. Biologically measuring the ratio of valid to invalid protein outputs would allow us to optimize the design process (and even if that ratio is low, it will still be orders of magnitude faster than natural evolution, a process which plays out on evolutionary timescales). To achieve both these ends, EASME will employ EA and GP models supplemented with ML where appropriate.

The authors are currently exploring several initial EASME projects and building an initial toolkit for open-source usage of EASME. An extended version of this paper is available on arXiv.org [6]. We plan to document the progress of the EASME field at https://aub.ie/easme. We welcome contributions from any researchers with an interest in this field.

**Key Points**

- The set of proteins produced by nature is minuscule compared to the search space of all possible proteins.
- AI algorithms capable of efficiently exploring this search space are now emerging.
- We propose a new AI framework, driven by evolutionary algorithms, capable of searching this space.
- The potential biotechnological impacts of this field are almost limitless.
